# Prevalence and predictors of self-reported multimorbidity and locomotor disability among the geriatric population in coastal South Karnataka, India

**DOI:** 10.1186/s12877-026-07466-y

**Published:** 2026-04-29

**Authors:** Yash Alok, Lahari U, Ramit Mahajan, Muralidhar M Kulkarni, Asha Kamath, Krishna Y, Shailaja Bhat, PSVN Sharma, Ranjitha S Shetty

**Affiliations:** 1 Department of Community Medicine, Kasturba Medical College, Manipal Academy of Higher Education, Manipal, India; 2 Department of Data Science, Prasanna School of Public Health, Manipal Academy of Higher Education, Manipal, India; 3 Department of Medical Rehabilitation Sciences, Audiology Program, College of Applied Medical Sciences, King Khalid University, Guraiger, Abha, 62529 Kingdom of Saudi Arabia; 4 Department of Ophthalmology, Kasturba Medical College, Manipal Academy of Higher Education, Manipal, India; 5 Department of Psychiatry, Kasturba Medical College, Manipal Academy of Higher Education, Manipal, India

**Keywords:** Aged, Activities of daily living, Chronic disease, Multimorbidity, Risk factors

## Abstract

**Background:**

The increasing proportion of geriatric individuals globally, particularly in low- and middle-income countries, including India, presents significant public health challenges and is accompanied by a growing burden of chronic illnesses and functional disabilities. This study aimed to estimate the prevalence of multimorbidity and locomotor disability among geriatric individuals in coastal South Karnataka and to examine their associations with sociodemographic factors.

**Methods:**

A community-based cross-sectional study was conducted from 2015-2017 across 15 villages in Udupi District, Karnataka. A total of 1832 geriatric individuals (≥60 years) were assessed and screened using pre-tested and validated structured questionnaires, and standard scales . Statistical analyses were conducted using Jamovi software, version 2.4.11. Logistic regression was used to analyse associations.

**Results:**

The prevalence of multimorbidity was 37.2%, with hypertension and diabetes being the most common conditions. Locomotor disability affected 30.2% of the population and was observed more commonly in females and those of advanced age. Locomotor disability is significantly associated with multimorbidity and hearing and visual impairments. Additionally, factors such as older age, single or not living with a spouse, lower levels of educational attainment, and middle socioeconomic status were also significantly associated with increased odds of locomotor disability. However, multimorbidity was found to be independently associated with only higher BMI.

**Conclusion:**

The study findings highlight a substantial burden of chronic diseases and functional limitations among the geriatric population in coastal Karnataka, necessitating comprehensive geriatric care strategies. Targeted interventions addressing modifiable risk factors, along with improved access to rehabilitative services and social security, are urgently needed to enhance elderly well-being and independence in this region.

## Introduction

The ageing of the population is a natural and global phenomenon that is accelerating. About one billion people worldwide were 60 years of age or older in the year2019, and estimates suggest that this number will rise even further by 2030. Two-thirds of this group is predicted to live in low- and middle-income nations by 2050. This demographic shift may strain their health systems [1], particularly in low- and middle-income countries (LMICs). In India, the number of geriatric individuals is expected to increase from 100 million in 2011 to 230 million by 2036, which may cause a shift in public health priority toward this issue [2, 3]. With increasing age, chronic diseases such as diabetes, hypertension, dementia, depression, musculoskeletal disorders, incontinence, visual and hearing impairments, and falls also become associated, leading to further difficulty in activities of daily living [4–7]. Therefore, with the increasing number of people falling under the bracket of the geriatric population, their age-responsive requirements for health and quality of life will also increase in demand through interventions and policies.

According to the World Health Organization (WHO), multimorbidity is defined as the presence of more than one chronic illness [8]. Globally, the prevalence of multimorbidity has been reported to be 37.2% [9]. In India, the prevalence of multimorbidity has ranged from 7.2% to 51.4% among various studies on older adults, with differences among urban and rural populations [10–14]. The study by Shenoy et al. from Karnataka has reported a prevalence ranging 9.4% to 36% in the age group from 61 to 90 years [15]. The prevalence of multimorbidity is often greater in the geriatric population, especially due to their restricted physical activity or locomotor disability, among other factors [16, 17]. The presence of multimorbidity further compounds their health needs and may require greater support from both caregivers and the health system, since it leads to a greater decline in the quality of life as well [14]. It has been reported that increasing number of co existing illnesses compound the negative effects of the illnesses on the disability of the person [18], which leads to a vicious cycle, and therefore multimorbidity may also act as a central predictor of functional decline. Locomotor disability is one of the major subgroups of disabilities affecting the elderly. Studies performed in India have demonstrated a prevalence of up to 25% for locomotor problems in the geriatric population [19].

Sociodemographic characteristics such as age, sex, marital status, educational attainment, and economic background have a major impact on the prevalence and consequences of multimorbidity and locomotor disability [19]. However, there are significant geographical differences, and limited research has been conducted specifically on the health requirements of the geriatric population in these regions. Also, due to the increased negative effects of increasing multimorbidity on locomotor disability, it becomes essential to study this association among other factors. Thus, the current study aims to ascertain the prevalence of multimorbidity and locomotor disability among the geriatric population of coastal South Karnataka and investigate their correlation with important sociodemographic factors.

## Materials and methods

### Study design and participants

A population-based cross-sectional study was conducted from March 2015 to March 2017 to study the profile of geriatric individuals in 15 villages across the Udupi District, Karnataka, India, with a total population of 50,000 across 7,357 families. The area is homogeneous in terms of occupation, socioeconomic status, and food habits. Healthcare services in the study area are provided by government and private agencies, including Kasturba Medical College and other local centres. All residents aged ≥ 60 years and not severely ill or bedridden were eligible for the study. Details on the sample size estimation and sampling strategy have been explained in an earlier publication [20]. The original study had objectives related to geriatric health assessment as the part of a major project. For sample size estimation, the disease with the least prevalence(dementia-3%) was chosen so that all other variables would be covered and a larger population could be assessed for improved generalizability [21].

### Data collection methods and instruments

Institutional ethical committee clearance was obtained to carry out the study. A team comprising trained personnel—a junior nurse and two medico social workers—visited the homes of the geriatric individuals. Each person was informed about the purpose of the visit and details of the study. They were assured that the confidentiality of the information provided would be maintained. Subjects were included in the study after written informed consent was obtained. During these visits, the trained team conducted personal interviews using predesigned and pretested questionnaire. The study tools were administered in the local language (Kannada). The questionnaire was translated and back-translated, and content validity was assessed independently by two subject experts. The questionnaire was pretested among 30 geriatric individuals from a similar population who were not included in the final analysis. Specific validated tools were used to assess visual and hearing impairments, as well as locomotor disabilities.

Sociodemographic characteristics of elderly individuals, such as age, sex, marital status, religion, educational qualifications, occupation, and income, were recorded. Age of the participants was ascertained from the Health and Demographic Surveillance System (HDSS), which maintains population data as family folders for each health centre under the Department of Community Medicine, Kasturba Medical College, Manipal. Personal habits, including a history of substance use, were also documented. Individuals who had smoked or consumed alcohol previously but had stopped the habit for ≥ 12 months prior to the survey and were not current users were classified as ex-smokers or ex-alcohol consumers. The socioeconomic status of the family was assessed using the modified Udai Pareek scale [22].

Height was measured in the standing position with participants barefoot against a wall to the nearest 0.5 cm. Body weight was recorded in kilograms using a standard weighing scale (Krups Weighing Scale, New Delhi, India) to the nearest 0.5 kg with light clothing. Body Mass Index (BMI) was calculated as weight (kg) divided by height (m²).

As defined by the WHO, multimorbidity refers to the presence of more than one chronic illness in an individual [8]. In our study, multimorbidity was defined as the presence of more than one self-reported chronic condition in an individual, specifically the coexistence of any two or more of the following: hypertension, diabetes, cardiovascular disease, stroke, cataract, osteoarthritis, low backache, asthma, thyroid disorder, and psychiatric illness. Visual acuity for distance vision was tested separately for both eyes via Snellen’s chart. Visual acuity was measured via the participant’s current refractive correction, if any. Alphabets (English/Kannada) were used for those who could read, and an E-type chart was used for those who could not. Distant visual acuity was measured in a standardized manner at a distance of 6 m [23, 24].

Both ears of the participants were examined via an otoscope by an audiologist to rule out any obstructive causes of hearing impairment, such as foreign bodies, impacted wax, and infections such as chronic suppurative otitis media. They were administered the Hearing Handicap Inventory for the Elderly Screening (HHIE-S) questionnaire to assess any hearing difficulties[25].

HHIE-S is a 10-item questionnaire. Responses are scored as Yes = 4 points, Sometimes = 2 points, and No = 0 points, giving a total score range of 0–40. Scores were interpreted as follows: 0–8 indicates no handicap, 10–24 indicates mild-to-moderate handicap, and 26–40 indicates severe handicap.

The Barthel Index of Activities of Daily Living (ADL) was used to assess the locomotor ability of the participants [26]. The index comprises 10 items addressing 10 different daily activities: bowels, bladder, grooming, toilet use, feeding, transfer, mobility, dressing, stairs, and bathing. Each item is given 2 to 4 responses and is scored on a scale of 0 to 3. The sum of an individual’s score can range from 0 to 20, with lower scores indicating increased disability. The items addressing feeding, grooming, dressing, and bathing indicate the ability to perform functions using upper limbs, whereas items such as mobility and stairs indicate the ability to perform some daily functions using lower limbs. For the purpose of logistic regression, the total Barthel Index score was dichotomized: a score of 20 (complete independence) was categorized as “No locomotor disability,” whereas scores ≤ 19 were categorized as “Yes – locomotor disability.”

### Ethical considerations

This study complied with the ethical guidelines of the Declaration of Helsinki. It was approved by the Kasturba Medical College and Kasturba Hospital Institutional Ethics Committee (Project approval no.255/2011; clinical trial no.: not applicable). Written informed consent was obtained from all participants before their inclusion in the study.

### Statistical analysis

The data were analysed using Jamovi version 2.4.11. Categorical variables are presented as frequencies and percentages. Continuous variables are primarily presented as medians and interquartile ranges(IQR); however mean and standard deviation (SD) are reported wherever they are more appropriate for data interpretation. Various associations were analysed via a logistic regression model, with the results presented as odds ratios (ORs) alongside 95% confidence intervals (CIs) and p-values. Multicollinearity was assessed via the variance inflation factor (VIF) before regression analyses. Variables with p values < 0.2 were included in the multivariate logistic regression analysis, where statistical significance was determined at *p* < 0.05.

## Results

### Socio demographic characteristics of the participants and prevalence of multimorbidity

The study included 1,832 geriatric individuals, with a mean age of 68.0 years (SD: 7.43). The mean age among males was 68.7 years, while females averaged 67.5 years. As presented in Table 1, hypertension was the most prevalent chronic condition, followed by diabetes and osteoarthritis. Females had more cases of osteoarthritis and thyroid disorders, whereas cardiovascular disease and stroke were more common in males. The median duration of illness varied across morbidities, with relatively longer durations noted for psychiatric and thyroid disorders. Overall, most participants with chronic diseases were receiving regular treatment. The prevalence of multimorbidity in the study population was 37.2%, and 45 individuals (2.45%) had more than three diseases. Among the male participants, 18.2% were smokers (42.3% were current smokers; 57.7% were ex-smokers), whereas a small proportion of females reported being smokers. In terms of smokeless tobacco use, such as snuff and chewing tobacco, more than 60% of the current users were females, whereas more than half of the ex-users were males. Alcohol consumption was predominantly male dominated, with 91.2% of current consumers and 96% of ex-consumers being male, whereas nonconsumption was considerably greater among females. 

**Table 1:** Gender distributions of the prevalence, duration, and treatment patterns of chronic diseases in the study population (*n* = 1832)

	Gender		Duration (years)	Treatment		Regular Treatment	
	Male (*n* = 756) No. (%)	Female (*n* = 1076) No. (%)	Median [IQR]	Yes	No	Yes	No
Hypertension (*n* = 1041)	429(41.2)	612(58.8)	6 [3–10]	1036 (99.5)	5 (0.5)	1027 (98.6)	14 (1.3)
Diabetes (*n* = 465)	219(41.3)	246(52.9)	7 [3–10]	452 (97.2)	13 (2.8)	448 (96.3)	17 (3.7)
Cardiovascular disease(*n* = 95)	51(53.7%)	44(46.3)	6 [3–10]	91 (95.8)	4 (4.2)	88 (92.6)	7 (7.4)
Stroke (*n* = 23)	18(78.3)	5(21.7)	4 [2.5–10]	21 (91.3)	2 (8.7)	21 (91.3)	2 (8.7)
Cataract (*n* = 14)	6(42.9)	8(57.1)	4 [2.5–10]	11 (78.6)	3 (21.4)	-	-
Osteoarthritis (*n* = 343)	106(30.9)	237(69.1)	4 [2–7]	319 (93.0)	24 (7.0)	298 (86.9)	45 (13.1)
Low backache (*n* = 159)	46(28.9)	113(71.1)	3 [2–6]	142 (89.3)	17 (10.7)	128 (80.5)	31 (19.5)
Asthma (*n* = 85)	32(37.6)	53(62.4)	5 [ 2–20]	81 (95.3)	4 (4.7)	76 (89.4)	9 (10.6)
Thyroid (*n* = 23)	4(17.4)	19(82.6)	7 [5-13.5]	22 (95.7)	1 (4.3%)	22 (95.7)	1 (4.3)
Psychiatric illness (*n* = 32)	7(21.9)	25(78.1)	8 [4–20]	32 (100.0)	0 (0.0)	31 (96.9)	1 (3.1)

*Abbreviations*: *IQR*Interquartile range

### Multimorbidity and its associations

In multivariable analysis, higher BMI remained independently associated with multimorbidity (AOR: 1.49; 95% CI: 1.20–1.85). Other sociodemographic variables were not statistically significant after adjustment as shown in Table 2.

**Table 2:** Association of the multimorbidity status of the elderly with their sociodemographic and anthropometric characteristics (*n* = 1832)

Characteristics	Category	Multimorbidity		UOR	AOR	*p* value
				(95% CI)	(95% CI)	
		Present	Absent			
		**No. (%)**	**No. (%)**			
Age	60–74	527(36.2)	929(63.8)	1	1	
	75–90	152(42.2)	208(57.8)	1.29(1.02–1.63)	1.15(0.89–1.50)	0.29
	> 90	4(25.0)	12(75.0)	0.59(0.19–1.83)	0.73(0.18–3.01)	0.666
Gender	Male	280(37.0)	476(63.0)	1		
	Female	403(37.5)	673(62.5)	1.09(0.84–1.23)	-	-
Education	Illiterate	184(37.2)	311(62.8)	0.96(0.77–1.19)	0.89(0.70–1.13)	0.346
	Up to 10 years of schooling	442(38.1)	718(61.9)	1	1	
	> 10 years of schooling	57(32.2)	120(67.8)	0.77(0.55–1.08)	0.74(0.50–1.10)	0.137
Religion	Hindu	555(36.2)	979(63.8)	0.87(0.61–1.25)	-	-
	Muslim	74(46.0)	87(54.0)	1.31(0.82–2.08)	-	-
	Christian	54(39.4)	83(60.6)	1	-	-
Marital status	Married	418(35.2)	771(64.8)	1	1	
	Single or not living with spouse	265(41.2)	378(58.8)	1.29(1.06–1.58)	1.25(0.99–1.57)	0.06
Occupation	Working	87(25.8)	250(74.2)	0.56(0.42–0.74)	0.47(0.13–1.78)	0.269
	Not working	242(41.4)	342(58.6)	1.13(0.90–1.42)	1.17(0.92–1.50)	0.208
	Retired	96(39.8)	145(60.2)	1.06(0.78–1.43)	1.29(0.90–1.83)	0.162
	Housewife	258(38.5)	412(61.5)	1	1	
Regular source of income	Yes	554(35.9)	991(64.1)	1	1	
	No	129(44.9)	158(55.1)	1.46(1.13–1.88)	1.31(0.98–1.74)	0.06
Income from salary	Yes	88(26.3)	247(73.7)	1	1.30(0.35–4.84)	0.698
	No	595(39.7)	902(60.3)	1.85(1.42–2.41)	1	
Pension	Yes	63(36.2)	111(63.8)	1	-	-
	No	620(37.4)	1038(62.6)	1.05(0.76–1.46)	-	-
Assets	Yes	154(34.6)	291(65.4)	1		
	No	529(38.1)	858(61.9)	1.65(0.93–1.46)	1.06(0.82–1.37)	0.652
Social scheme	Yes	181(41.4)	256(58.6)	1	1	
	No	502(36.0)	893(64.0)	0.80(0.64–0.99)	0.82(0.63–1.07)	0.146
BMI	< 18.50	57(24.2)	179(75.8)	0.57(0.42–0.78)	**0.50(0.36–0.70)**	**< 0.001**
	18.50-24.99	348(36.1)	617(63.9)	1	1	
	>=25	254(43.9)	324(56.1)	1.39(1.13–1.72)	**1.49(1.20–1.85)**	**< 0.001**
Socio Economic status*(*n* = 1818)	High	59(35.8)	106(64.2)	1	1	
	Middle	456(35.5)	827(64.5)	0.99(0.71–1.39)	1.03(0.71–1.49)	0.897
	Low	163(44.1)	207(55.9)	1.42(0.97–2.07)	1.52(0.99–2.33)	0.055

Statistically significant results are presented in bold

*UOR *unadjusted odds ratio,* AOR *adjusted odds ratio, *CI *confidence intervals,* BMI *body mass index

*Socioeconomic status details were incomplete in the remaining forms

**Fig. 1:**
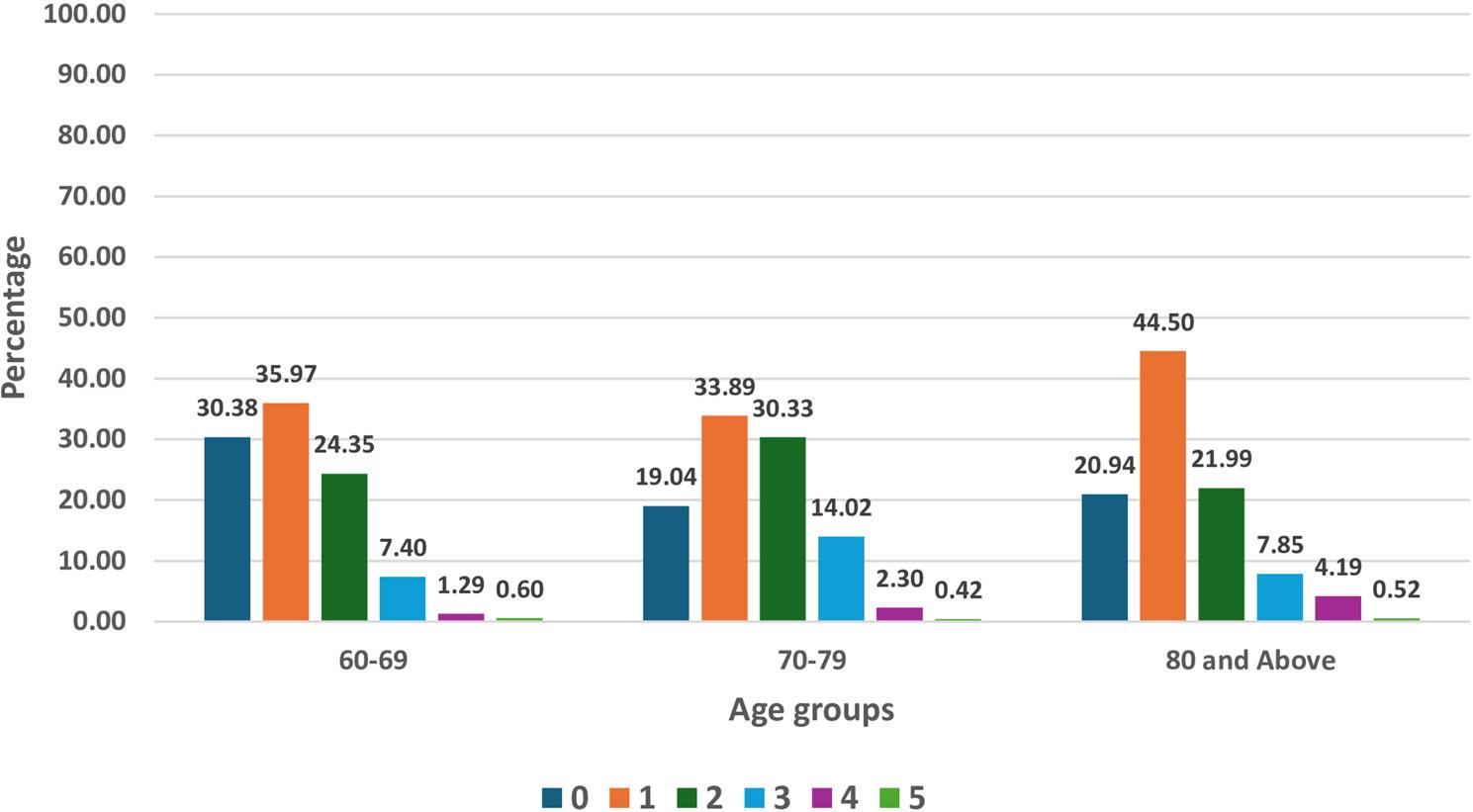
Association of count of illnesses with age group

Figure 1 shows the association of count of illnesses among various age groups. It was observed that with advancing age, the number of diseases in one individual increased significantly(Chi square-53.97, df-10, *p*<0.001).

### Locomotor disability, its distribution and associations

**Fig. 2:**
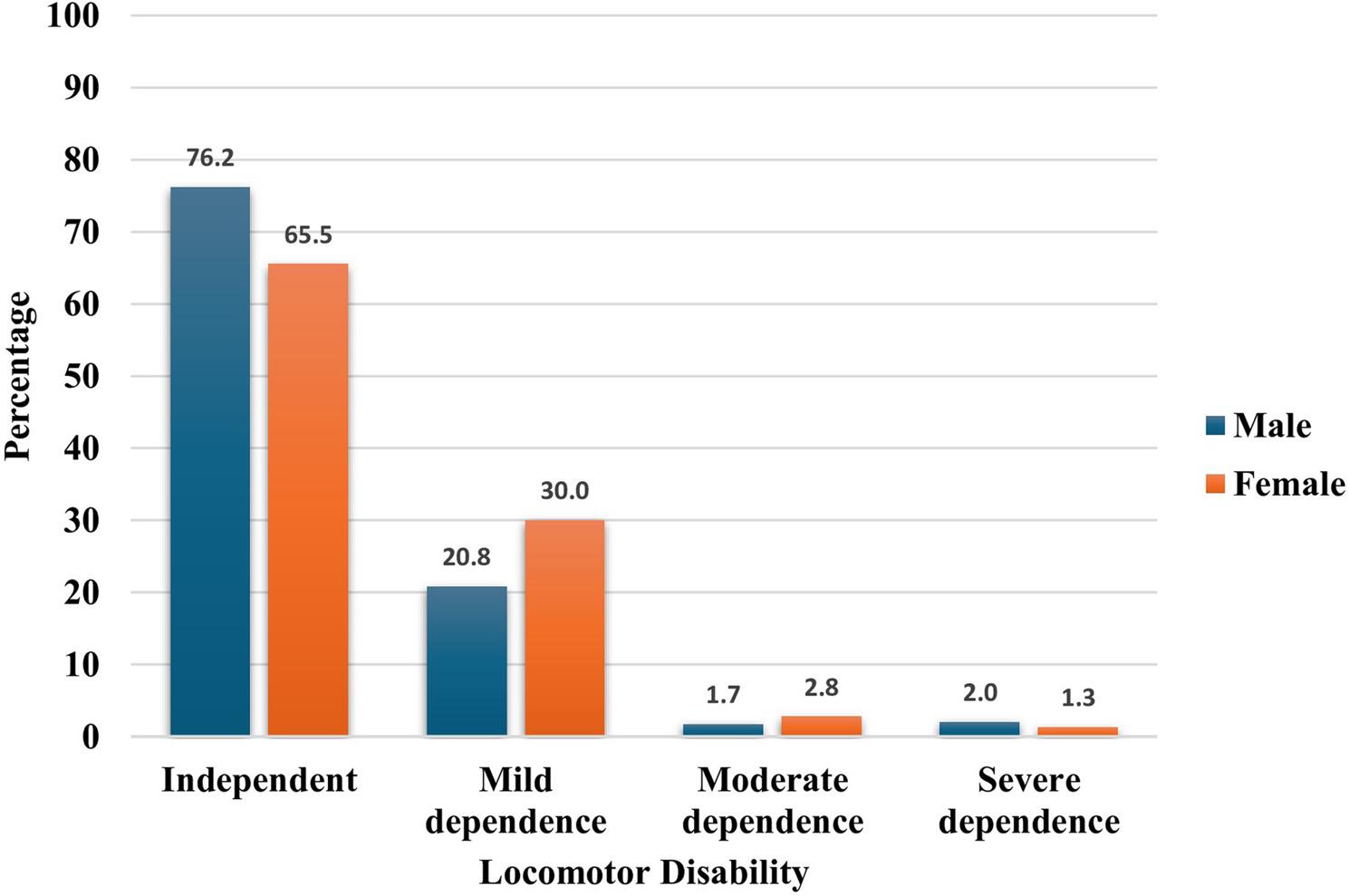
Gender-wise distribution of locomotor disability in the study population (*n*=1816)

As shown in Fig. 2, the bar chart illustrates locomotor disability among males and females. Overall, 27.4% of males and 34.5% of females were found to be dependent for their activities of daily living, and most of the dependent females were in the mild dependence category based on the Locomotor Disability Scale.

Multivariable analysis revealed that individuals aged 75--90 years (AOR: 3.09, 95% CI: 2.32--4.12), those aged >90 years (AOR: 8.65, 95% CI: 1.54--48.75), those with lower literacy (AOR: 1.56, 95% CI: 1.19--2.04), Hindus by religion (AOR-2.04, 95% CI: 1.19--3.48) or Muslim (AOR: 2.59, 95% CI: 1.36--4.95), those who were single or not living with a spouse (AOR: 1.67, 95% CI: 1.26--2.22) and those with middle socioeconomic status (AOR: 1.60, 95% CI: 1.01--2.54) had higher odds of experiencing locomotor disability. Conversely, individuals with high literacy, who were retired, and who did not have assets or social security schemes were found to have lower odds of locomotor disability, as indicated in Table 3. 

**Table 3:** Association of locomotor ability with sociodemographic and anthropometric characteristics (*n* = 1816)

Characteristics	Category	Locomotor Disability		UOR	AOR	*p* value
		Yes	No	(95% CI)	(95% CI)	
		No. (%)	No. (%)			
Age	60–74	330(22.8)	1115(77.2)	1	1	
	75–90	206(57.7)	151(42.3)	4.61(3.61–5.88)	**3.09(2.33–4.11)**	**< 0.001**
	> 90	12(85.7)	2(14.3)	20.27(4.52–91.04)	**8.65(1.54–48.75)**	**0.014**
Gender	Male	183(24.4)	567(75.6)	1	1	
	Female	365(34.2)	701(65.8)	1.61(1.31–1.99)	1.09(0.77–1.54)	0.693
Education	Illiterate	221(45.5)	265(54.5)	2.33(1.87–2.91)	**1.56(1.19–2.04)**	**< 0.001**
	Up to 10 years of schooling	304(27.4)	849(73.6)	1	1	
	> 10 years of schooling	23(13.0)	154(87.0)	0.42(0.26–0.66)	**0.54(0.32–0.94)**	**0.029**
Religion	Hindu	474(31.1)	1048(68.9)	2.01(1.28–3.14)	**2.04(1.19–3.48)**	**0.009**
	Muslim	49(31.0)	109(69.0)	2.00(1.15–3.46)	**2.59(1.36–4.95)**	**0.004**
	Christian	25(18.4)	111(81.6)	1	1	
Marital status	Married	919(77.9)	260(22.1)	1	1	
	Single or not living with spouse	349(54.8)	288(45.2)	2.92(2.37–3.59)	**1.71(1.28–2.27)**	**< 0.001**
Occupation	Working	41(12.2)	295(87.8)	0.25(0.17–0.36)	1.00(0.23–4.32)	0.999
	Not working	228(39.6)	348(60.4)	1.18(0.94–1.49)	1.25(0.91–1.73)	0.174
	Retired	42(17.5)	198(82.5)	0.38(0.26–0.55)	**0.61(0.37–0.99)**	**0.044**
	Housewife	237(35.7)	427(64.3)	1	1	
Regular source of income	Yes	476(31.1)	1057(68.9)	1	1	
	No	72(25.4)	211(74.6)	1.46(1.13–1.88)	0.79(0.56–1.11)	0.177
Income from salary	Yes	40(12.0)	294(88.0)	1	1	
	No	508(34.3)	974(65.7)	1.85(1.42–2.41)	2.40(0.56–10.24)	0.238
Pension	Yes	39(22.4)	135(77.6)	1	-	-
	No	509(31.0)	1133(69.0)	1.05(0.76–1.46)	-	-
Assets	Yes	153(34.4)	292(65.6)	1	1	
	No	395(28.8)	976(71.2)	1.17(0.93–1.46)	**0.46(0.34–0.62)**	**< 0.001**
Social security scheme	Yes	235(54.5)	196(45.5)	1	1	
	No	313(22.6)	1072(77.4)	0.80(0.64–0.99)	**0.46(0.35–0.61)**	**< 0.001**
BMI	< 18.50	94(40.2)	140(59.8)	1.78(1.32–2.40)	1.27(0.91–1.80)	0.157
	18.50-24.99	263(27.4)	698(72.6)	1	1	
	>=25	156(27.0)	421(73.0)	0.98(0.79–1.24)	1.22(0.94–1.60)	0.125
Socioeconomic status	High	38(23.0)	127(77.0)	1	1	
	Middle	400(31.2)	882(68.8)	1.52(1.04–2.22)	**1.60(1.01–2.54)**	**0.045**
	Low	110(29.8)	259(70.2)	1.42(0.93–2.17)	1.53(0.90–2.59)	0.114

Statistically significant results are presented in bold

*UOR *unadjusted odds ratio, *AOR *adjusted odds ratio, *CI *confidence intervals, *BMI *Body mass index

Furthermore, locomotor disability was also found to be significantly associated with multimorbidity, hearing impairment, and visual impairment, as indicated in Table 4.

**Table 4:** Association of locomotor disability with multimorbidity and sensory impairments (*n* = 1816)

Characteristics		Locomotor Disability		UOR (95% CI)	AOR (95% CI)	p value
		Yes No. (%)	No No. (%)			
Multimorbidity	Present	241(35.6)	436(64.4)	1.50(1.22–1.84)	**1.46(1.19–1.80)**	**< 0.001**
	Absent	307(27.0)	832(73.0)	1	1	
Hearing impairment Score (HHIE-S)	≥ 10	97(56.4)	75(43.6)	3.42(2.48–4.71)	**3.26(2.36–4.50)**	**< 0.001**
	< 10	451(27.4)	1193(72.6)	1	1	
Visual impairment	Yes	540(30.8)	1213(69.2)	3.06(1.45–6.47)	**2.67(1.26–5.67)**	**0.010**
	No	8(12.7)	55(87.3)	1	1	

Statistically significant results are presented in bold

*UOR *unadjusted odds ratio, *AOR *adjusted odds ratio, *CI *confidence intervals, *HHIE-S *Hearing Handicap Inventory for Elderly Screening

## Discussion

Multiple morbidities were present in more than one-third of the participants in the study. Similar results were obtained in the study by Patel et al., possibly because of their similar risk factor profiles and sociodemographic characteristics [27]. Globally, similar trends were observed (37.2%), with a higher prevalence in developed countries in the study by Chowdhury et al., possibly due to differences in demographic structure [9]. Increasing age was associated with increasing counts of multimorbidity, similar to the findings reported in the study by Kumar and Singh [28]. Multimorbidity was significantly associated with higher BMI. Individuals with a BMI ≥ 25 had greater odds of multimorbidity than those with a normal BMI. Obesity is a well-established risk factor for conditions such as hypertension, diabetes, dyslipidemia, and osteoarthritis. This association is largely attributed to contributing factors such as low physical activity or lack of mobility. Studies by Romano et al., and Boro and Saikia also support these findings [29, 30]. Additionally, being single and not living with one’s spouse was linked to higher odds of having multimorbidity. It could be hypothesized that the loss of spousal support may have negatively affected health-seeking behavior, medication adherence, diet, and mental well-being, leading to a greater disease burden in single individuals. In our study, hypertension was the most common chronic disease reported, affecting over half of the study participants, with a notably higher prevalence among females. This aligns with previous community-based studies in India, such as those by Farron et al. (63.2% of hypertensive patients, with 48% of the diagnosed hypertensive patients being women), Kothavale et al. (53% of female hypertensive patients) and other developing countries, such as China, Ghana and Mexico, in the study by Lloyd-Sherlock et al.^4^ (55% of female hypertensive patients) [31–33]. The factors associated with hypertension reported in other studies were age, sex, obesity and substance abuse [33]. The preponderance of hypertension among females could be explained by women having increased awareness of hypertension [34], thereby being more likely to undergo screening and being detected for hypertension [35]. as well as the fact that there is a higher trend of hypertension after the age of 65 years in women and poor control [36].

Diabetes mellitus was the second most common condition, affecting nearly one-fourth of the geriatric population in our study. This was in contrast to other studies, such as the one by Das and Kar, which reported that the prevalence of diabetes mellitus was 12%, and another study by Medhi et al. (7.5%) [37, 38]. The difference in prevalence of our study and other studies could be explained by the variation in socioeconomic factors and study methodologies, such as sample size and sampling techniques. As the trend of noncommunicable diseases is increasing in the country, with a simultaneous increase in the population of elderly individuals, population-based and high-risk screening and early initiation of treatment for noncommunicable diseases should be prioritized to mitigate the upcoming public health issue.

In our study, cardiovascular disease and stroke were more prevalent among males. In contrast, studies by Kundu and Kundu, Shahanawaz et al. and Banerjee et al. reported that female sex was associated with an increased risk of cardiovascular disease [39–41]. However, other Indian studies, such as that by Kodali and Bhat and Kalita et al., have shown that men are at greater risk of cerebrovascular events, possibly due to greater exposure to risk factors such as smoking, alcohol consumption, and occupational stress [42, 43]. The patterns are, in global comparison, similar to European and American trends [44, 45]. Conversely, musculoskeletal conditions such as osteoarthritis and thyroid disorders were more common among females in the present study, which is similar to findings in Indian studies performed by Marwaha et al. and Chibber et al. [46, 47] This could be attributed to postmenopausal changes, longer life expectancies, and nutritional deficiencies [48].

In the present study, psychiatric illnesses had the longest median duration of illness (8 years), suggesting chronic underdiagnosis or delayed intervention for mental health conditions in geriatric individuals, since they could have been diagnosed a long time after the onset of symptoms, owing to factors such as stigma, lack of care from caregivers, inaccessibility of services etc. [49]. This has also been reported in other studies, such as the one by Tiwari and Pandey [50], and in international studies, such as the one by Ojagbemi et al. [51]. This finding indicates that integrating mental health services into primary geriatric care is a priority.

Encouragingly, treatment adherence was high (> 90%) for major chronic illnesses, reflecting relatively high awareness and possibly better access to healthcare. This finding is higher as compared to the results obtained in studies by Sharma et. al(80.3%), Khaiser et al.(57%),and Angadi et al.(58.5%) [52–54]. The differences observed could be due to the differences in socio demographics, higher level of awareness among the study population and variation in tools used for measurement of regularity of treatment.Substance use patterns showed marked gender differences. Smoking and alcohol consumption were predominantly male behaviors, with more than 90% of current and former users being men. Moreover, the use of smokeless tobacco (snuff and chewing) was more prevalent among females in our study population. These patterns are consistent with broader national trends reported in studies such as the Global Adult Tobacco Survey-2 (GATS), which also reported higher rates of smokeless tobacco use among Indian women, albeit lower than in men [55]. Similar trends internationally have also been observed in other developing countries, such as Nepal and Bangladesh [56]. Sociocultural factors such as the acceptance of society for women consuming smokeless tobacco but not smoking tobacco may explain these gendered patterns of substance use. The high prevalence of ex-users among males suggests a trend toward cessation, possibly due to factors that prompt behavioral change, such as the onset of noncommunicable diseases. These findings highlight the importance of gender-sensitive tobacco and alcohol cessation programs targeted at specific geriatric populations.

Locomotor disability affected approximately one-third of the population, particularly females. These findings were similar to the findings from the study by Maroof et al., possibly due to reduced muscle mass in women, leading to increased exposure to disabling conditions such as osteoporosis and arthritis [11]. Mild dependence was more common than severe dependence, suggesting that early interventions focused on physical rehabilitation could reverse or mitigate disability progression. Age was the most significant determinant of locomotor disability, with individuals above 90 years having an eightfold greater risk than those aged 60–74 years. These findings are consistent with those of other studies, such as that by Maroof et al., which may indicate that the prevalence of disability increases exponentially with age due to increased physiological decline [19]. The association of higher odds of locomotor disability with the Hindu and Muslim religions than with the Christian religion may reflect disparities in sociocultural factors. This pattern may also be influenced by the larger proportion of Hindus in the population, although further research is needed to substantiate these associations. Education appeared to be a strong protective factor. Those with ≥ 10 years of schooling had significantly lower odds of locomotor disability, with similar results as those reported in the study by Maroof et al., but the results were not statistically significant [19]. This could be due to the influence of education on health literacy, access to resources, and better management of health conditions, thereby preventing functional decline. Socioeconomic factors, such as not having a regular source of income from assets and social security schemes, were found to have lower odds of locomotor disability. This association could also be due to a greater proportion of older individuals without any source of income in the present study.

Multimorbidity, hearing impairment, and visual impairment were found to be independent predictors of locomotor disability. Hearing impairment increased the odds of locomotor disability by more than three times, whereas visual impairment more than doubled the odds. Sensory impairments can severely compromise balance, spatial navigation, and social interaction, thereby promoting physical decline and functional dependency. These results highlight the need for routine vision and hearing screening as part of routine geriatric evaluation. Early detection and rehabilitation (e.g., provision of hearing aids, cataract surgeries) could play a crucial role in maintaining mobility and independence in elderly populations.

These findings highlight the pressing need for integrated geriatric care in India, in accordance with the International Care for Older People (ICOPE) framework of the WHO [57]. The global trend is transitioning from disease-specific care to function-oriented and person-centered paradigms, particularly in aging populations. The National Program for the Health Care of Elderly Individuals in India aligns with these guidelines and focuses on providing care to elderly individuals as a part of primary care, but its implementation and coverage need strengthening. These results also align with the Sustainable Development Global (SDG) 3- Good health and well-being; SDG-10- Reduced inequalities within and among countries [58]. The detection of a significant prevalence of multimorbidity and functional impairment among older people and methods to resolve them focus on this goal, especially with its objectives pertaining to universal health coverage and the reduction of premature mortality from noncommunicable diseases. Therefore, the implications of this study for public health practice involve emphasis on comprehensive screening for risk factors and chronic noncommunicable diseases, as well as for visual and auditory impairments among geriatric population. Health promotion focusing on modifiable risk factors like obesity, screening for body mass index (BMI) and waist-hip ratio in primary care for the elderly, and routine physiotherapy/occupational therapy for locomotor disability integrated into primary care will help to reduce the burden of disease. Provision of subsidized healthcare and expansion of social security schemes for the elderly may help to reduce burden of cost of healthcare. The enforcement of insurance programs such as Ayushman Bharat for the elderly may help to achieve better healthcare access and utilization for the same [59].

The strengths of the study include the use of a large sample of community-dwelling elderly individuals, enhancing the generalizability of the findings to similar rural and coastal regions in India. Both disease prevalence and functional status were systematically assessed via standardized instruments.

However, the study has certain limitations. Its cross-sectional design restricts any causal interpretations. Disease status and substance use were based on self-reports, introducing potential recall and reporting biases. Disease prevalence was also based on self-reports and included only already diagnosed cases, which limited the validity of the study, as there might have been underreported cases, highlighting a higher burden than obtained in the study. Certain conditions, such as psychiatric illnesses, may have been underreported due to stigma and other associated factors. The exclusion of severely ill or bedridden patients may have added to the underestimation of the true prevalence of severe disability and multimorbidity in the target population. Furthermore, the study population was from a coastal region with specific sociocultural characteristics, which may not be representative of elderly populations in other regions of India. Near vision assessment using subjective correction was not performed. Inclusion of this assessment could have enhanced the robustness of visual impairment evaluation.

## Conclusion

This study highlights the substantial burden of multimorbidity, visual and auditory impairments, and locomotor disability among elderly people in coastal South Karnataka, with clear associations with increased age, higher BMI, single marital status, low educational status, and lower financial security. Prioritization of holistic screening for chronic noncommunicable diseases and risk factors, as well as for functional and visual and hearing impairments, may be a helpful approach. Preventive strategies like health promotion targeting modifiable risk factors such as obesity, intensification of screening of BMI assessment and waist hip ratio in geriatric primary care, and promoting education attainment, as well as providing subsidized healthcare for elderly individuals, could significantly reduce the burden of disability. Policymakers may need to explore the expansion of social security schemes for the elderly, ensure universal health coverage for elderly populations, and strengthen community-based rehabilitation programs focusing on vision, hearing, and mobility support for the elderly, in addition to social support systems.

## Data Availability

The datasets generated and/or analysed during the current study are not publicly available owing to ethical considerations and restrictions imposed by the data sharing policy of the affiliated institution. Reasonable requests for data access may be considered by the corresponding author in accordance with institutional guidelines and approvals.

## Abbreviations

ADL: Activities of Daily Living
AOR: Adjusted odds ratio
BMI: Body mass index
CI: Confidence interval
GATS: Global Adult Tobacco Survey
HHIE-S: Hearing Handicap Inventory for the Elderly Screening
IQR: Interquartile range
LMICs: Low- and middle-income countries
NCDs: Non-Communicable Diseases
OR: Odds ratio
SES: Socioeconomic Status
UOR: Unadjusted odds ratio
VIF: Variance inflation factor
WHO: World Health Organization
